# Supplementary material for: Taxonomic revision of Eoalligator (Crocodylia, Brevirostres) and the paleogeographic origins of the Chinese alligatoroids
Source: PeerJ. 2016 Aug 24;4:e2356. doi: 10.7717/peerj.2356 (PMC5012266; doi:10.7717/peerj.2356)
Supplement: Supplemental Information 1 [file peerj-04-2356-s001.docx]

**Supplementary Information**

The coding of new taxa:

“*Eoalligator_chunyii*”

0010????????1010????????????????????????????????0021?2????????????00?01?01?????0?????????????????????1???????????????01?1????????0??1?1?????0??????01??0?0010110???1?????1???1?00?003????????11

*Protoalligator_huiningensis*

??????????????????????????????????????????????110021????????111&2???????????????00100?000??1101000?1??????????????????????????????001?1?1??0???????????????????????????????????????????????????2?

*Asiatosuchus nanlingensis*

????????????????????????????????????????????????0?2????????0?02???0???1?01????00??????????????????????????????????????????????????????????????????????????????????????????????????????????????1?

*Krabisuchus siamogallicus*

????????????????????1??0?????????10??11???1???11110??1?????00?110100?01001????00010???00?01020?00100???????????1000001011100001000?0110100?1?00?0?0??2001??1?112????????????????10??1??????????

Maoming specimen

??????????????????????????????????????????????????????????????????????????????????????????1?????0?????????0????????????????????0001?1????????????????200???1???????????????????????????????????

The codings of modified and new characters:

Character 74:

1???1?000?00?00?100?00????1111???1111111111111???1???11?0?11??101??111?111??111?0?00??010?00?11??11??3?1?3

Character 190:

?????????????????????0???1??????????22????????????????????2222??11021???????????1?????????????????????2???
